# Whole-Genome Sequencing of a Human Clinical Isolate of the Novel Species *Klebsiella quasivariicola* sp. nov.

**DOI:** 10.1128/genomeA.01057-17

**Published:** 2017-10-19

**Authors:** S. Wesley Long, Sarah E. Linson, Matthew Ojeda Saavedra, Concepcion Cantu, James J. Davis, Thomas Brettin, Randall J. Olsen

**Affiliations:** a Center for Molecular and Translational Human Infectious Diseases Research, Department of Pathology and Genomic Medicine, Houston Methodist Research Institute and Houston Methodist Hospital, Houston, Texas, USA; b Department of Pathology and Laboratory Medicine, Weill Cornell Medical College, New York, New York, USA; c Computing, Environment and Life Sciences, Argonne National Laboratory, Argonne, Illinois, USA; d Computation Institute, University of Chicago, Chicago, Illinois, USA

## Abstract

In a study of 1,777 *Klebsiella* strains, we discovered KPN1705, which was distinct from all recognized *Klebsiella* spp. We closed the genome of strain KPN1705 using a hybrid of Illumina short-read and Oxford Nanopore long-read technologies. For this novel species, we propose the name *Klebsiella quasivariicola* sp. nov.

## GENOME ANNOUNCEMENT

Members of the genus *Klebsiella* are a common cause of human morbidity and mortality (1, 2). In a recent study of 1,777 *K. pneumoniae* isolates producing extended-spectrum β-lactamase (ESBL), which were recovered from patients in our health care system, we identified strain KPN1705 as a distinct outlier in the phylogenetic analysis (3, 4). It shared a common branch with *K. variicola*, yet was as distant from the *K. pneumoniae*, *K. quasipneumoniae*, and *K. variicola* reference genomes as they were from each other. A more complete comparison of KPN1705 to other *Klebsiella* spp. has been described elsewhere (5).

The genome of strain KPN1705 was previously described using Illumina short-read data (3). We sequenced the genome of strain KPN1705 to closure using a one-dimensional ligation sequencing kit, an R9.4 flow cell, and an Oxford Nanopore Technologies MinION Mk-Ib sequencer. We used the Unicycler hybrid assembler version 0.4.0 to achieve complete closure of the genome and plasmids using a combination of the Illumina and Oxford Nanopore data (6). The KPN1705 chromosome is 5,540,188 bp, and three plasmids were identified, pKPN1705-1 (240,771 bp), pKPN1705-2 (97,896 bp), and pKPN1705-3 (67,851 bp). These plasmids carry a diverse array of replicons and antimicrobial resistance genes. Six intact phage regions were predicted in the core chromosome using PHASTER, consisting of 359 coding sequences in 322.7 kb of core chromosomal sequence (7). We assessed the antimicrobial gene content of KPN1705 and determined that it carries the LEN-24 β-lactamase on its chromosome, similar to what is commonly found in *K. variicola*. This further contributed to our decision to call this novel species *K. quasivariicola* sp. nov. Strain KPN1705 also carried the gene encoding the SHV-30 ESBL enzyme on plasmid pKPN1705-3. Genes encoding KPC, OXA, CTX-M, TEM, and NDM-1 were not detected.

To determine if strains similar to KPN1705 had been previously reported, we determined its multilocus sequence type (ST). Results revealed that it is a single locus variant of ST-1155, with three single nucleotide polymorphisms in the *infB*_110 allele. A search of publicly available databases found one previous report of an ST-1155 *Klebsiella* isolate, which was a description of a novel *Klebsiella* sp. named strain 10982 (8). Strain 10982 was recovered from a perianal swab collected on an intensive care unit patient in Maryland in 2005 as part of a study of AmpC-mediated antimicrobial resistance (8).

The discovery of this novel clade of *Klebsiella* spp., the *K. quasivariicola* sp. nov., represents yet another *Klebsiella* sp. capable of causing serious human infections. Importantly, when novel strain 10982 was first described, its pathogenic potential was unclear. The novel strain KPN1705 was recovered from a wound culture. In addition, the detection of multiple antimicrobial resistance genes, including an SHV ESBL enzyme, increases its virulence potential. These data provide new insight into the natural history and pathogenesis of *Klebsiella* organisms. Improved diagnostic methods or widespread use of whole-genome sequencing of clinical isolates may be necessary to ensure timely and appropriate identification of these pathogens. Together, these whole-genome sequence data suggest that KPN1705 and strain 10982 represent a novel *Klebsiella* sp., and we propose the name *Klebsiella quasivariicola* sp. nov.

### Accession number(s).

The GenBank accession numbers for the KPN1705 closed genome and plasmids are CP022823 to CP022826.
